# Seizure-like activity in hyaluronidase-treated dissociated hippocampal cultures

**DOI:** 10.3389/fncel.2013.00149

**Published:** 2013-09-12

**Authors:** Maria Vedunova, Tatiana Sakharnova, Elena Mitroshina, Maya Perminova, Alexey Pimashkin, Yury Zakharov, Alexander Dityatev, Irina Mukhina

**Affiliations:** ^1^Laboratory for Brain Extracellular Matrix Research, Lobachevsky State University of Nizhny NovgorodNizhny Novgorod, Russia; ^2^Cell Technology Group, Nizhny Novgorod State Medical AcademyNizhny Novgorod, Russia; ^3^Molecular Neuroplasticity Group, German Center for Neurodegenerative DiseasesMagdeburg, Germany; ^4^Medical Faculty, Otto-von-Guericke University of MagdeburgMagdeburg, Germany

**Keywords:** extracellular matrix, hyaluronic acid, AMPA receptors, L-type Ca^2+^ channels, neuronal network, microelectrode array, Ca^2+^ imaging, seizure

## Abstract

The extracellular matrix (ECM) plays an important role in use-dependent synaptic plasticity. Hyaluronic acid (HA) is the backbone of the neural ECM, which has been shown to modulate α-amino-3-hydroxyl-5-methyl-4-isoxazolepropionate (AMPA) receptor mobility, paired-pulse depression, L-type voltage-dependent Ca^2+^ channel (L-VDCC) activity, long-term potentiation and contextual fear conditioning. To investigate the role of HA in the development of spontaneous neuronal network activity, we used microelectrode array recording and Ca^2+^ imaging in hippocampal cultures enzymatically treated with hyaluronidase. Our findings revealed an appearance of epileptiform activity 9 days after hyaluronidase treatment. The treatment transformed the normal network firing bursts and Ca^2+^ oscillations into long-lasting “superbursts” and “superoscillations” with durations of 11–100 s. The changes in Ca^2+^ transients in hyaluronidase-treated neurons were more prominent then in astrocytes and preceded changes in electrical activity. The Ca^2+^ superoscillations could be suppressed by applying the L-VDCC blocker diltiazem, whereas the neuronal firing superbursts could be additionally suppressed by 6-cyano-7-nitroquinoxaline-2,3-dione as an antagonist of AMPA/kainate receptors. These results suggest that changes in the expression of HA can be epileptogenic and that hyaluronidase treatment *in vitro* provides a robust model for the dissection of the underlying mechanisms.

## INTRODUCTION

The extracellular matrix (ECM) plays an important role in regulating use-dependent synaptic plasticity. Distinct aggregates of ECM molecules surround cell bodies and proximal dendrites of some central neurons, forming so-called perineuronal nets (PNNs; Dityatev et al., 2010). These nets are heterogeneous in their structure and composition and are composed of molecules produced by both neurons and astrocytes (Dityatev et al., 2010), such as hyaluronic acid (HA), chondroitin sulfate proteoglycans of the aggrecan family, tenascin-R and link proteins (Bruckner et al., 2000; Galtrey and Fawcett, 2007; Frischknecht and Seidenbecher, 2008). HA is a large, negatively charged, non-branched polymer composed of repeated disaccharides of glucuronic acid and *N*-acetylglucosamine. Recently, HA has been shown to affect both α-amino-3-hydroxyl-5-methyl-4-isoxazolepropionate (AMPA) glutamate receptor mobility and paired-pulse modulation in hippocampal cultures (Frischknecht et al., 2009). Another study established a mechanistic link between HA and long-term potentiation (LTP) by showing that removing HA by hyaluronidase (Hyase) suppresses L-type voltage-dependent Ca^2+^ channel (L-VDCC)-mediated currents in hippocampal slices, reduces Ca^2+^ transients in postsynaptic dendrites and spines, and specifically abolishes an L-VDCC-dependent component of LTP (Kochlamazashvili et al., 2010). Furthermore, the injection of Hyase before contextual fear conditioning impairs the formation/retention of fear memories. A mathematical modeling study highlighted that the ECM may function as a memory substrate by showing that remodeling of ECM may lead to a bistability in which two different stable levels of average firing rates can coexist in a spiking network (Kazantsev et al., 2012).The multiple roles played by the ECM in healthy brain neuroplasticity suggest that it could be an important factor for pathogenic plasticity associated with epileptogenesis (Dityatev, 2010), which results in the transformation of normal neuronal network activity into spontaneous recurrent epileptiform discharges (seizures; Robert et al., 1998). Although accepted to be important, the exact contribution of the multiple structural and functional pathological forms of plasticity in hippocampal circuit alteration during epileptogenesis remains to be elucidated. For most forms of epilepsy, it is unknown how the hippocampus becomes hyperexcitable and how hyperexcitable cells integrate into a pathologically functioning circuit, resulting in the generation of spontaneous recurrent seizures. In recent years, microelectrode array (MEA) and optical imaging technologies have developed rapidly, providing new options to perform long-term, detailed analysis of neural circuitry dynamics.

In this study, we used these technologies to describe the development of epileptiform activity following Hyase treatment *in vitro*. Our findings revealed that Hyase destructed the ECM of PNNs, surrounding cell bodies and the proximal dendrites of parvalbumin (PV)-expressing interneurons, and induced a slow development of seizure-like activity. The treatment transformed normal network spiking bursts into long-lasting “superbursts” and caused the appearance of neuronal and astrocytic Ca^2+^ “superoscillations.” Seizure-like activity in Hyase-treated cultures persisted for at least 9 days and could be suppressed by an L-VDCC blocker but not by an NMDA receptor antagonist. These results suggest that changes in the expression of HA can be epileptogenic and the underlying mechanisms may involve changes in Ca^2+^ oscillations.

## MATERIALS AND METHODS

### CELL CULTURES

Hippocampal cells were dissociated from embryonic mice (on embryonic day 18) and plated with a high initial density of approximately 9000 cells/mm^2^ on MEAs (Alpha MED Science, Japan) pre-treated with the adhesion promoting molecule polyethyleneimine (Sigma P3143). We used so high-density to mimic tissue conditions and have long-lasting recordings of network activity. The cultures develop a complex pattern of activity as previously described (Pasquale et al., 2008). C57BL6J mice were killed by cervical vertebra dislocation, according to the protocols approved by the National Ministry of Public Health for the care and use of laboratory animals and by the Bioethics Committee of the Nizhny Novgorod State Medical Academy. Embryos were removed and decapitated. The entire hippocampi were dissected under sterile conditions. Hippocampi were cut in Ca^2+^- and Mg^2+^-free phosphate-buffered saline (PBS-minus). After enzymatic digestion for 25 min by 0.25% trypsin (Invitrogen 25200-056) at 37°C, cells were separated by trituration (10 passes) using a 1 ml pipette tip. After being passed, the solution was centrifuged at 1500 × *g* for 2 min, and the cell pellet was immediately re-suspended in Neurobasal medium (Invitrogen 21103-049) with 2% B27 (Invitrogen 17504-044), 0.5 mM L-glutamine (Invitrogen 25030-024), and 5% fetal calf serum (PanEco K055) (NBM1). The dissociated cells were seeded in a 40 μl droplet covering the center of the culture dish on a 1 mm^2^ electrode region of the MEA, forming a dense monolayer (Potter and DeMarse, 2001). After the cells had adhered (usually within 2 h), the dishes were filled with 0.8 ml of NBM1. After 24 h, the plating medium was replaced by a medium containing Neurobasal medium with 2% B27, 1 mM L-glutamine, and 0.4% fetal calf serum (NBM2) without any antibiotics or antimycotics. Glial growth was not suppressed because glial cells are essential for long-term culture maintenance. One half of the medium was changed every 2 days. The cells were cultured under constant conditions of 37°C, 5% CO_2_, and 95% air at saturating humidity in a cell culture incubator (MCO-18AIC, Sanyo).

Phase-contrast images of the cultures were taken weekly to record the culture status using a DMIL HC (Leica, Germany) inverted microscope with a 10×/0.2Ph1 objective. Experiments were performed when the cultures reached the 17th day *in vitro* (DIV).

### ELECTROPHYSIOLOGICAL METHODS

Extracellular potentials were recorded simultaneously through 64 planar indium tin-oxide (ITO) platinum black electrodes with the integrated MED64 system (Alpha MED Science, Japan). MEAs were 8 × 8 (64) with a 50 μm × 50 μm electrode size and a 150 μm spacing, and the sampling rate was 20 kHz/channel.

All of the signal analyses and statistics were performed using custom-made software (Matlab^®^).

### SPIKE DETECTION

The detection of recorded extracellular spikes was based on threshold calculation using the signal median:

(1) $$T=N_{S\sigma ,\sigma }=median(\frac{\left|x\right|}{0.6745}),$$

where *x* is the band-pass-filtered (0.3–8 kHz) data signal, σ is an estimate of the standard deviation of the signal without spikes (Quiroga et al., 2004), and *N*_S_ is a spike detection coefficient determining the detection threshold (Pimashkin et al., 2011). In signal processing the threshold estimation based on the median of the signal in a form of Eq. 1 is less dependent on the frequency of the spikes than the estimation based on standard deviation. Coefficient 0.6745 in Eq. 1 is used for normalization of the median of the absolute signal to standard deviation. *N*_S_**= 4 was used for all data, resulting in a reliable detection of spikes with amplitudes greater than 20 μV. The minimal interspike interval was set to 1 ms. Detected spikes were plotted in raster diagrams.

### SPONTANEOUS ACTIVITY ANALYSIS

#### Small burst detection

To analyze the effect of Hyase on neural network activity, we recorded spontaneous bursting activity for 10 min. To detect small bursts, we calculated the total spiking rate (TSR) accounting for the total number of spikes from all electrodes within 50 ms time bins. A fast appearance of a large number of spikes over multiple electrodes in a small (50 ms) time bin was used as a criterion for small burst appearance (for more details, see Pimashkin et al., 2011). Spontaneous activity in the culture consists of a basal stochastic activity observed in a fraction of cells and short bursting episodes. The basal activity was consisted of a spike trains (~1 spike per 10–100 ms). To detect bursts we used threshold detection based on the statistical characteristics of the spontaneous activity TSR(t). Burst threshold was set to be T_Burst_ =0.1× σ _TSR_ , where σ _TSR_ is standard deviation of TSR(t). To exclude the basal activity, the burst detection threshold coefficient was empirically set to 0.1, giving the best estimate for the burst initiation and ending points recognized in the raster diagram. Simulation of bursts with the frequency of occurrence up to 5 Hz revealed that the estimated duration of bursts was biased less than 10% of real values. Statistical analysis of the bursting activity characteristics was performed by analysis of variance (ANOVA) tests (*p* < 0.05).

#### Superburst detection

**FIGURE 1:**
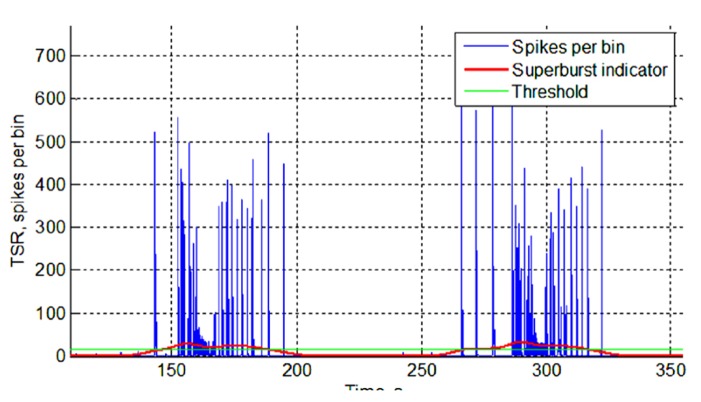
**Superburst detection.** TSR (blue), total spiking rate over all electrodes in every consecutive 50 ms bin. The superburst indicator (red) reflects the cross-correlation of the TSR with a 50-s long Gaussian window. The estimated threshold for superburst detection is shown in green (for details, see Spontaneous Activity Analysis).

Superbursts in the electrical activity recorded from the multielectrode arrays were detected as follows. First, we defined a Gaussian function with an effective width equal to 50 s. Next, we iteratively moved that function from the beginning of the recording to the end in 10 ms time steps and calculated a cross-correlation of the function with the TSR. The resulting cross-correlation indicated how much of the synchronized activity (bursts) was recorded in the 10 s window (**Figure 1**). To detect superbursts in the spiking activity, we applied threshold detection, in which the threshold was estimated as the spiking superburst detection accuracy coefficient multiplied by the standard deviation of the calculated cross-correlation. The superburst detection accuracy coefficient was found empirically and was equal to 0.4. All time points that crossed the threshold were defined as the beginnings and endings of the superbursts.

### Ca^2+^ imaging

#### Dye loading

Oregon Green 488 BAPTA-1 AM (OGB-1) (0.4 μM; Invitrogen) was dissolved in dimethylsulfoxide (DMSO) with 4% pluronic acid and NBM2 (pH 7.4) and gassed with 95% O_2_ and 5% CO_2_ at 35.5°C. After 40-min incubation for near-full absorption of the OGB-1 molecules by the cultured cells, the exposed culture was washed for 15 min with NBM2 in the absence of dye. Additionally, the exposed dissociated cultures were also loaded with the astrocyte-specific indicator sulphorodamine SR101 (10 μM; Invitrogen S359; Nimmerjahn et al., 2004).

#### Optical techniques

A confocal laser scanning microscope, Zeiss LSM 510 (Germany), with a W Plan-Apochromat 20×/1.0 objective was used to investigate the spontaneous activity of the neuronal and astrocytic network. Cytosolic Ca^2+^ was visualized via OGB-1 excitation with the 488 nm line of Argon laser radiation and emission detection with a 500–530 nm filter, while astrocytes were visualized with SR101, which was excited by 543 nm radiation from a He–Ne laser and detected with the use of a 650–710 nm filter for emission. Time series of 256 × 256-pixel images with a 420 μm × 420 μm field of view were recorded at a rate of 4 Hz. A confocal pinhole of 1 Airy unit ensured an axial optical slice resolution of 1.6 μm.

#### Image analysis

Quantitative evaluation of Ca^2+^ transients was performed off-line using custom-made software in C++ Builder. Cell regions from fluorescent images were manually selected. The Ca^2+^ fluorescence for each cell in each frame was calculated as the average fluorescence intensity (F, relative units from 0 to 255) of the pixels within the defined cell region. Single Ca^2+^ signals were found using the following algorithm. First, each trace from all of the cells was filtered by averaging two neighboring points in the sample set. Next, we calcuated a simple derivative of the signal by calculating a difference between each pair of consequent points. The pulses were found from the derivative of the trace using a threshold detection algorithm. The threshold was estimated as the detection accuracy coefficient multiplied by the standard deviation of the derivative of the trace. Suprathreshold points on the derivative of the trace were taken as the beginnings and endings of the pulses. The detection accuracy coefficient was empirically set to 0.45.

To detect superoscillations, we filtered the signal with a low-pass elliptic filter (0.2 Hz) that removed any regular short calcium pulses. Then, we calculated the derivative of the filtered signal. Each point of the derivative was estimated as an average of the differences of the 20 subsequent pairs of points. This definition allowed us to clearly visualize the superoscillation beginnings and endings. Next, we applied a threshold detection algorithm to detect these superoscillations. The threshold was estimated as the superoscillation detection accuracy coefficient multiplied by the standard deviation of the derivative of the trace. All of the time points that crossed the threshold were defined as the beginnings and endings of the superoscillation. The superoscillation detection accuracy coefficient was empirically set to 0.8.

### IMMUNOCYTOCHEMISTRY

#### Staining

The cultured cells were fixed for 15 min in 4% formaldehyde containing phosphate-buffered saline (PBS; pH 7.4), washed in PBS and permeabilized for 30 min with 0.1% Triton X-100 (Sigma 93443-100ML) and 2% bovine serum albumin (BSA). Subsequently, the cells were incubated for 2 h at room temperature in PBS containing 1% BSA and the appropriate mixture of the primary antibodies: rabbit polyclonal anti-aggrecan (AB1031, Millipore) to stain PNNs and chicken anti-microtubule-associated protein 2 (MAP2) (AB15452, Millipore) to stain neurons. After washing in PBS, the cell cultures were incubated for 2 h at room temperature with the following secondary antibodies: goat anti-rabbit conjugated Alexa Fluor 555 (A21429, Invitrogen) and goat anti-chicken conjugated Alexa Fluor 647 (A 21245, Invitrogen). The immunostained cultures were examined under a confocal laser scanning microscope (Zeiss LSM510, Germany), with a W Plan-Apochromat 20×/1.0 objective. The laser intensity, gain and offset were held constant for each analysis. Quantitative evaluation was performed using Image J (Research Service Branch, NIH).

#### Quantification of PNNs

The number of MAP2-positive neurons bearing a PNN was determined on cultures that were double-labeled with anti-MAP2 and anti-aggrecan antibodies. In five 420 μm × 420 μm fields of view, we sampled all MAP2-immunopositive hippocampal neurons and assessed the presence of PNNs surrounding each individual neuron (*N* = 5 cultures/DIV).

### PHARMACOLOGICAL AGENTS

Drugs were applied to cultures using a pipette. One group received 100 μl of hyaluronidase (Hyase, from *Streptomyces hyalurolyticus*, Sigma H1136; 75 U/ml), and the second group received Hyase that had been boiled for 30 min (control group). The Hyase was dissolved in PBS, added on the 17th DIV to the cell cultures and incubated at 35.5°C for 1 day, i.e., the culture medium was changed on the 18th DIV.

Pharmacological analysis was performed on DIV 20. AMPA and NMDA glutamate receptors were blocked by 10 μM 6-Cyano-7-nitroquinoxaline-2.3-dione (CNQX; Sigma, C127) and 10 μM 3-(2-carboxypiperazin-4-yl)propyl-1-phosphonic acid (CPP; Sigma, C104), respectively. L-VDCCs were blocked by 10 μM diltiazem.

### STATISTICAL ANALYSIS

All data quantification is presented as the mean ± standard error of the mean (SEM). Statistical analysis was performed using a two-way ANOVA implemented in the SigmaPlot 11.0 program (Systat Software Inc.). Student–Newman–Keuls (SNK) was used as a post hoc ANOVA test. The difference between groups was considered significant if the *p* value was less than 0.05.

## RESULTS

**FIGURE 2:**
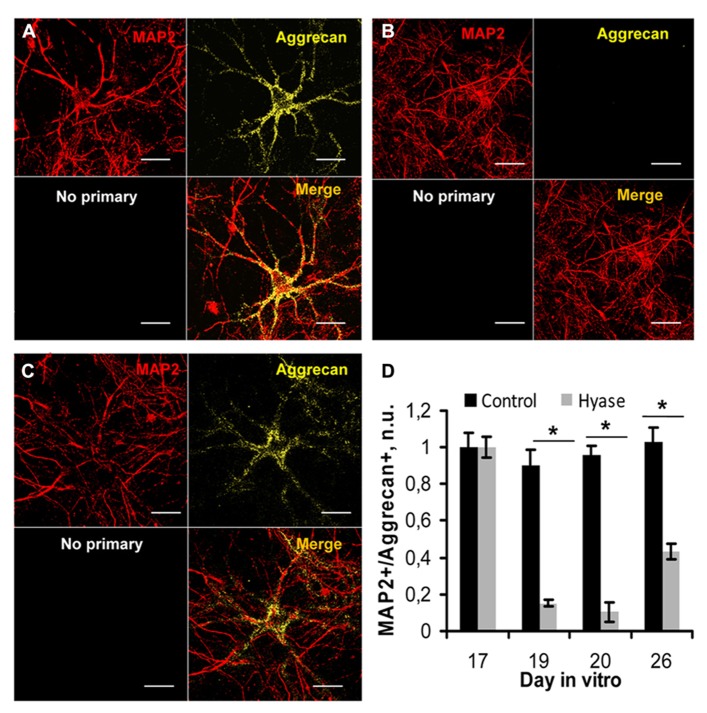
**The effect of hyaluronidase on PNNs in the primary hippocampal cultures (A–C)**. PNNs, stained by aggrecan antibodies (yellow), envelop hippocampal cultured neurons, which were labeled by MAP2 antibodies (red). Hippocampal neurons in the control **(A)** and Hyase-treated primary hippocampal cultures **(B,C)** 2 days after Hyase treatment, i.e., on DIV 19 **(B)**, and 9 days after Hyase treatment, i.e., on DIV 26 **(C)**. “No primary” panels (black) represent immunostainings done without primary antibodies **(A–C)**. Scale bar: 20 μm. **(D)** The fraction of MAP2-positive neurons bearing aggrecan-positive PNNs at different time intervals after Hyase treatment (ANOVA; *p < 0.05; *N* = 5 cultures/DIV). The data are from five independent culture preparations. The values shown were normalized by the mean fractions of PNNs in the control and Hyase groups on the 17th DIV. n.u., normalized units; MAP2, microtubule-associated protein 2; Hyase, hyaluronidase.

Hyaluronic acid is the backbone of neural ECM, which is enriched in PNNs. To remove HA and study its role in neural network activity, we treated hippocampal cultures with Hyase. Because aggrecan has been shown to be a key component of the ECM of PNNs (Giamanco et al., 2010), we used aggrecan immunostaining to characterize the efficacy of the treatment. **Figure 2** shows hippocampal neurons labeled by anti-MAP2 antibodies and PNNs labeled by anti-aggrecan antibody in the control treatment (**Figure 2A**) and after Hyase treatment (**Figures 2B, C**). The treatment was done on DIV 17 when the number of PNNs in the control group reached a steady-state level (**Figure 2D**). Counting the PNNs in the control and Hyase-treated primary hippocampal cultures revealed a strong reduction in the fraction of aggrecan-immunopositive neurons 2 (ANOVA *p* = 0.001), 3 (ANOVA *p* < 0.001), and 9 (*p* = 0.019) days after Hyase treatment (**Figure 2D**). Between days 3 and 9, however, there was an increase in the fraction of PNNs (ANOVA *p* = 0.037), suggesting PNN re-formation during this time interval.

**FIGURE 3:**
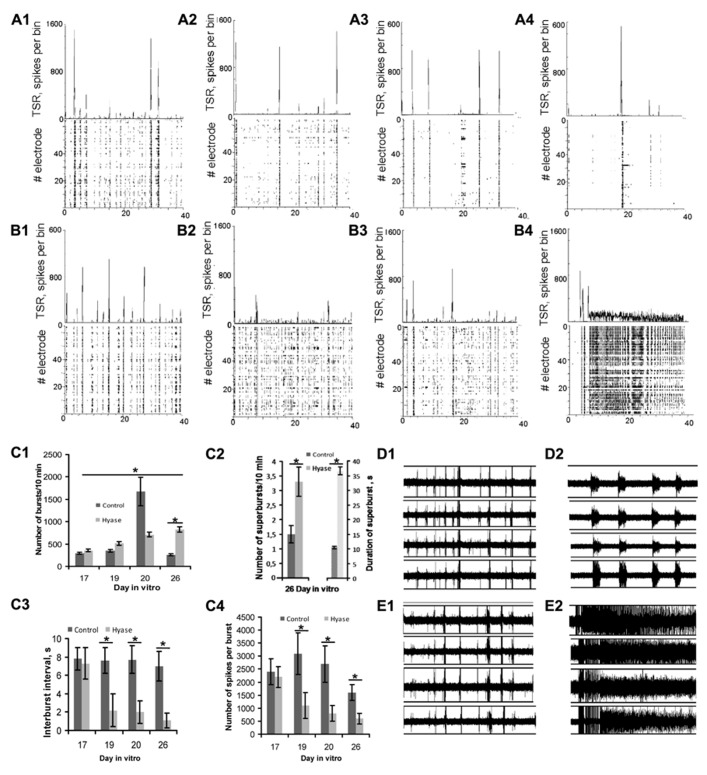
**Raster plots of electrical spiking activity over 64 electrodes (lower rows) and total spike rate diagram (upper row) in primary hippocampal cultures (A1–A4,B1–B4)**. Hippocampal neuronal network activity in intact cultures on the 17th DIV **(A1,B1)**. **(A2,A3)** Neuronal network activity in control cultures on DIV 19 **(A2)**, DIV 20 **(A3)**, and DIV 26 **(A4)**. **(B2–B4)** Development of activity in Hyase-treated cultures 2 days after Hyase treatment on DIV 19 **(B2)**, 3 days after Hyase treatment on DIV 20 **(B3)**, and 9 days after Hyase treatment on DIV 26 **(B4)**. In the latter panel, a seizure-like superburst is shown. **(C1)** Number of network bursts per 10 min after Hyase treatment (ANOVA; *p < 0.05; *N* = 5). **(C2)** Number and duration of network superbursts per 10 min on day 9 after Hyase treatment. **(C3)** Interburst interval (s) after Hyase treatment (ANOVA; *p < 0.05; *N* = 5). **(C4)** Number of spikes per burst after Hyase treatment (ANOVA; *p < 0.05; *N* = 5). **(D1,D2,E1,E2)** Spike recording from 4 electrodes of MED64 probes: **(D1)** intact culture on the 17th DIV; **(D2)** the same culture on the 26th DIV, 9 days after boiled Hyase application; **(E1)** intact culture on DIV 17; **(E2)** the same culture on DIV 26, 9 days after Hyase application. TSR, total number of spikes over all electrodes per 50 ms bin; Hyase, hyaluronidase.

Previous studies demonstrated that spontaneous spiking activity appears in high-density dissociated hippocampal cultures after 8–10 days of development on MEAs (Wagenaar et al., 2006). On DIV 16, dissociated culture activity became stabilized. To establish whether the Hyase treatment changes the spontaneous network activity, we analyzed raster plots of electrical spiking activity and spike rate diagrams (**Figures 3A1–A4,B1–B4**). We revealed significant changes in spontaneous spiking activity 9 days after Hyase treatment. Short network bursts with duration of less than 10 s (**Figures 3D1, D2**) were transformed into seizure-like superbursts of activity with durations of 15–35s (**Figures 3C2, E1, E2**). These changes in the spontaneous spiking activity of Hyase-treated cultures appeared on days 5–7 following the treatment and were observed during the next 4 days.

Statistical analysis revealed that the superbursts (**Figures 3C1–C4**) consist of a large number of network bursts, which are characterized by a low number of spikes per burst and a short interburst interval. Moreover, the general number of network bursts per 10 min increased significantly from day to day after Hyase treatment (19 DIV: 513.3 ± 36; 20 DIV: 712 ± 54; 26 DIV: 827 ± 62, ANOVA; *p* < 0.05 compared to DIV 17; *N* = 5), and this parameter was significantly different between the Hyase and control groups on DIV 26 (Control: 257 ± 21, ANOVA *p* < 0.05; *N* = 5).

In the control group on the 26th DIV, we recorded short superbursts with a frequency of 1.5 ± 0.3 per 10 min and a mean duration of 10.5 ± 0.5 s. After Hyase treatment, we observed long superbursts with a frequency of 3.3 ± 0.5 per 10 min and a mean duration of 36.5 ± 1.4 s (ANOVA; *p* < 0.05; *N* = 5).

**FIGURE 4:**
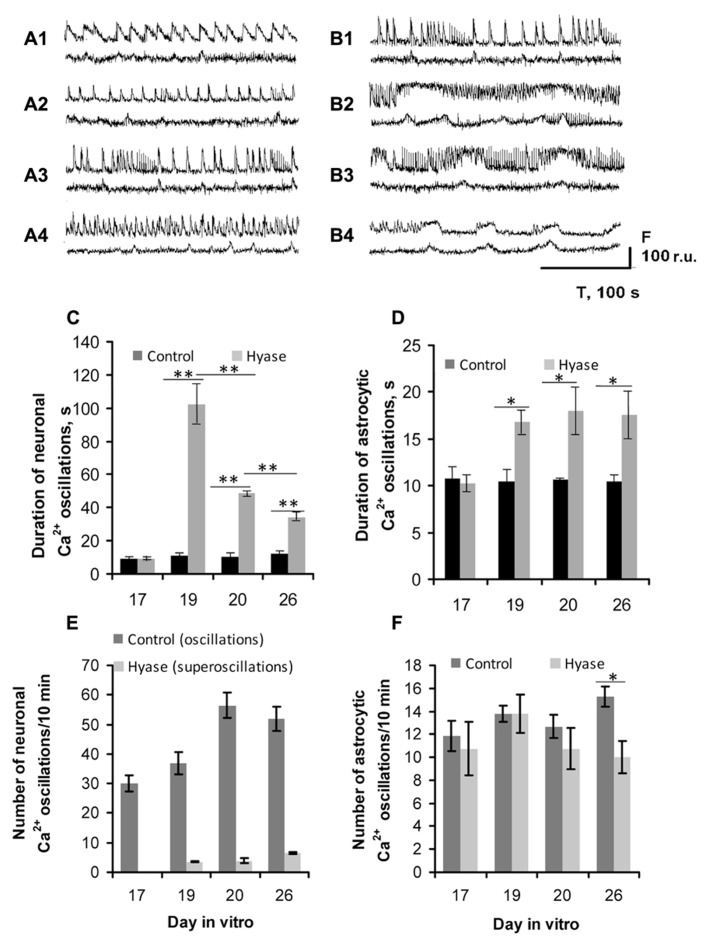
**Hyaluronidase-induced Ca^2+^ superoscillations in the neuronal (upper curves) and glial (lower curves) networks in dissociated hippocampal cultures. (A,B)** Spontaneous Ca^2+^ oscillation recordings from: **(A1)** DIV 17, intact; **(A2)** DIV 19, control; **(A3)** DIV 20, control; **(A4)** DIV 26, control; **(B1)** DIV 17, intact; **(B2)** DIV 19, 2nd day after Hyase application, showing the appearance of neuronal Ca^2+^ superoscillations with maximal duration; **(B3)** DIV 20, 3rd day after Hyase application, some astrocytic Ca^2+^ transients demonstrate synchronization with the neuronal superoscillations; **(B4)** DIV 26, 9th day after Hyase application, the neuronal Ca^2+^ superoscillations are shorter but the astrocytic oscillations are longer compared to DIV 19. **(C)** Time-course of the changes in the neuronal Ca^2+^ oscillation duration after Hyase application. **(D)** Time-course of the changes in the astrocytic Ca^2+^ oscillation duration after Hyase application. **(E)** Number of neuronal and **(F)** astrocytic Ca^2+^ oscillations after Hyase application. Neuronal Ca^2+^ superoscillations appear 2 days after the Hyase treatment on DIV 19, and there is no such activity in the control cultures. There is no difference between the Control and Hyase groups in the frequency of astrocytic Ca^2+^ oscillations before DIV 26, but they differ on DIV 26 (Control 15.75 ± 0.89, Hyase 10.3 ± 0.9, ANOVA; *p* < 0.05; *N* = 5). The astrocytic Ca^2+^ oscillations after Hyase treatment become less frequent but longer. *p < 0.05; **p < 0.001 indicate significant differences by ANOVA, *N* = 5.

Spontaneous Ca^2+^ oscillations appear in dissociated hippocampal cultures beginning on DIV 7 (unpublished observation). On the DIV 17, a large number of neurons have similar patterns of Ca^2+^ oscillations, which are quite distinct from the patterns of Ca^2+^ oscillations in astrocytes (**Figures 4A1, B1**). Both neuronal and astrocytic patterns of Ca^2+^ activity were fairly stable and did not change significantly up to the 26th DIV (**Figures 4A2–A4**). The duration of the neuronal oscillations was approximately 6 s (**Figures 4C, D**) and the duration of the astrocytic Ca^2+^ oscillations was approximately 10 s on DIV 17–26. The Hyase treatment significantly changed the spontaneous Ca^2+^ activity (**Figures 4B2–B4, 4C–F**), and already on the second day after Hyase application, we observed the appearance of neuronal Ca^2+^ superoscillations with a duration of 94.78 ± 12.33 s (**Figure 4C**). The superoscillation duration then showed a significant decrease (20 DIV: 50.95 ± 1.25 s; 26 DIV: 28.54 ± 2.71 s; ANOVA *p* < 0.001 for both days compared to the 19th DIV; *N* = 5). Additionally, an increase in the duration of the astrocytic Ca^2+^ oscillations was observed after Hyase application (19 DIV: 16.78 ± 1.21 s; 20 DIV: 18.03 ± 2.48 s; 26 DIV: 17.75 ± 2.53 s; ANOVA; *p* < 0.05 for all days compared to DIV 17; *N* = 5). We also observed that some astrocytes synchronized their Ca^2+^ activity with that of neurons starting from the 2nd day after the Hyase treatment (**Figures 4B2–B4**).

**FIGURE 5:**
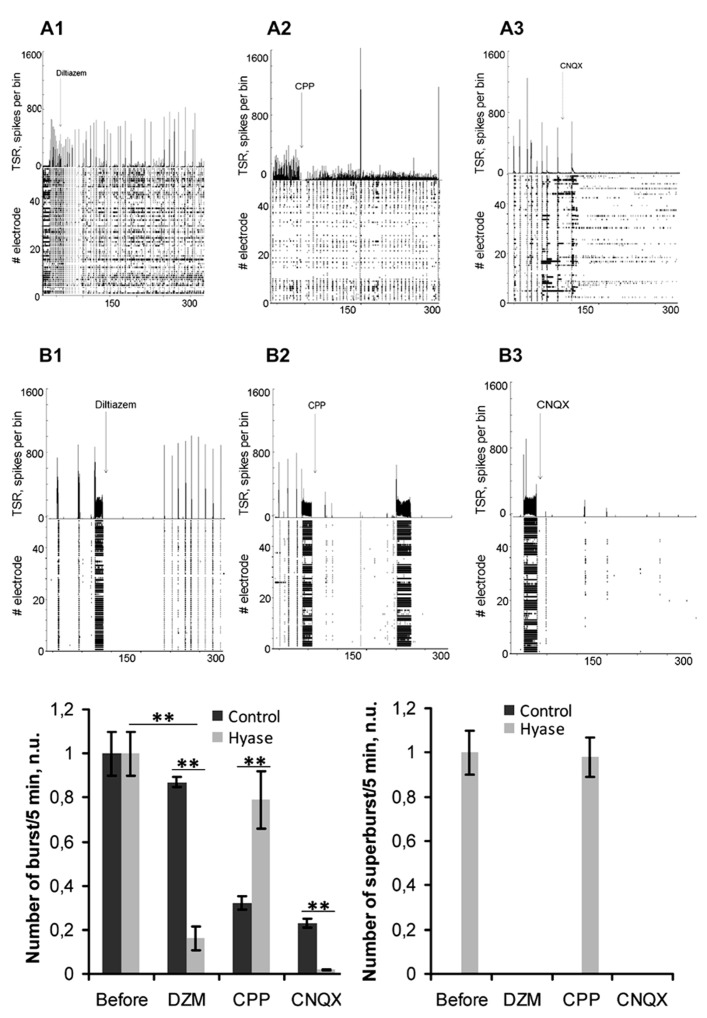
**Pharmacological suppression of Hyase-induced superbursts of neuronal spiking activity. (A,B)** Spontaneous burst recordings on the 26th DIV, 5 min after blocker application: **(A1)** Control group treated with diltiazem; **(A2)** Control group treated with CPP; **(A3)** Control group treated with CNQX; **(B1)** Hyase group treated with diltiazem; **(B2)** Hyase group treated with CPP; **(B3)** Hyase group treated with CNQX. **(C,D)** Number of bursts and superbursts after blocker application. CPP: (±)-3-(2-carboxypiperazin-4-yl)propyl-1-phosphonic acid, 10 μl; CNQX, 6-Cyano-7-nitroquinoxaline-2,3-dione, 10 μl; Hyase, hyaluronidase; DZM, diltiazem, 10 μM. Mean ± SEM are shown, **p < 0.001 indicate significant differences by ANOVA, *N* = 5.

**FIGURE 6:**
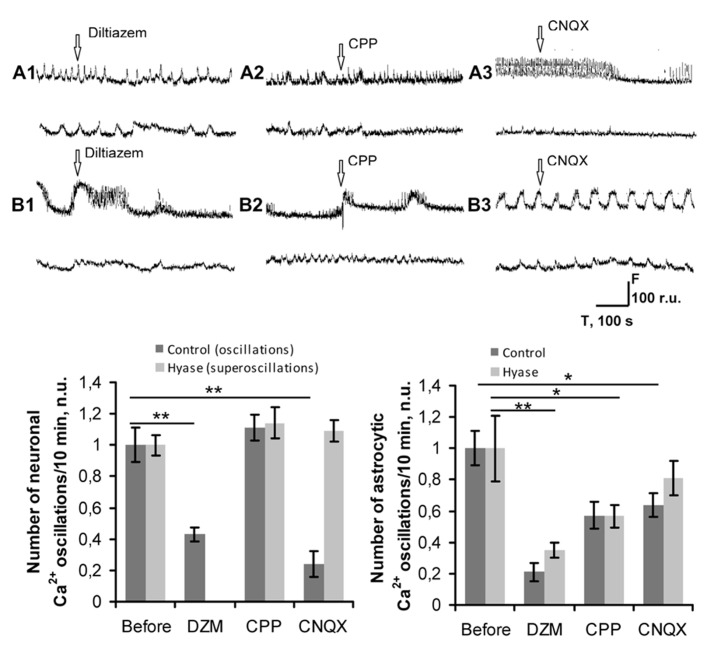
**Pharmacological suppression of Hyase-induced long-lasting Ca^2+^ oscillations in neuronal networks. (A,B)** Spontaneous Ca^2+ ^oscillations recordings on DIV 26, 5 min after blocker application. Upper curves show neuronal transients, lower curves represent astrocytic transients. **(A1)** Control group treated with diltiazem; **(A2)** Control group treated with CPP; **(A3)** Control group treated with CNQX; **(B1)** Hyase group treated with diltiazem; **(B2)** Hyase group treated with CPP; **(B3)** Hyase group treated with CNQX. **(C)** Blockage of L-VDCCs decreases the neuronal Ca^2+ ^ oscillations in control neurons and decreases both Ca^2+ ^oscillations in astrocytes and Ca^2+ ^superoscillations in Hyase-treated neurons. **(D)** Number of astrocytic Ca^2+^ oscillations. There is no difference between the control and Hyase-treated groups. CPP, (±)-3-(2-carboxypiperazin-4-yl)propyl-1-phosphonic acid, 10 μl; CNQX, 6-cyano-7-nitroquinoxaline-2,3-dione, 10 μl; Hyase, hyaluronidase; DZM, diltiazem, 10 μM. Mean ± SEM values are shown, *p < 0.05; **p < 0.001 indicate significant differences by ANOVA, *N* = 5.

Next, we attempted to block the Hyase-induced superbursts of spiking activity and Ca^2+^ superoscillations in the neuronal and glial networks (**Figures 5 and 6**). Seizure-like elevations in the burst activity of Hyase-treated cultures could be suppressed within 5 min after treatment by the L-VDCC blocker diltiazem (**Figures 5B1, C, D**) but not by the NMDA receptor antagonist CPP (**Figures 5B2, C, D**). An AMPA/kainate receptor antagonist, CNQX, also suppressed superbursts. Neuronal Ca^2+^ superoscillations could also be blocked by diltiazem (**Figure 6B1**, upper curve; **Figure 6C**) but not by CPP (**Figure 6**). Diltiazem also significantly reduced the number of astrocytic Ca^2+^ oscillations after the Hyase treatment (**Figure 6B1**, lower curve; **Figure 6D**).

## DISCUSSION

Here, we demonstrated that a Hyase treatment, which results in the removal of HA as the backbone of the neural ECM, leads to a slow development of epileptiform activity in cultured hippocampal neurons. These data provide a new *in vitro* model of epileptogenesis that can be easily induced and studied using MEA technology and optical imaging to characterize the processes underlying epileptogenesis.

Many animal models of epilepsy already exist. The majority involve experimental induction of a brain injury through the administration of a chemoconvulsant or electrical stimulation, both of which induce an episode of seizures. Subsequent injury-induced plasticity includes the appearance of mossy fiber sprouting (Parent and Lowenstein, 1997), an increased rate of neurogenesis in the dentate gyrus (Parent et al., 1997), increased activity of microglia, and an induction of reactivity in astrocytes (Wetherington et al., 2008). In addition to cell death and anatomical changes in neurons and glia, seizures induce significant functional plasticity in surviving hippocampal neurons, including alterations in the function of Ca^2+^- and hyperpolarization-activated mixed cationic channels (Shah et al., 2004; Jung et al., 2010) and K^+^ channels (Bernard et al., 2004), changes in neurotransmitter receptors, including GABA_A_ receptors (Pathak et al., 2007), glutamate receptors (Doherty and Dingledine, 2001), chloride transporters (Pathak et al., 2007) and excitatory amino acid transporters (Crino et al., 2002; Eid et al., 2008).

Thus, multiple mechanisms could underlie epileptogenesis. Because Hyase treatment leads to a disruption of PNNs around PV-expressing GABAergic interneurons, it is plausible to suggest that one of the causes of Hyase-induced epileptiform activity is an imbalance in excitation and inhibition due to impaired PV interneuron function. These cells represent the most frequent group of inhibitory cells, and they are widely distributed across the brain (Karetko and Skangiel-Kramska, 2009). However, PNN removal with another enzyme, chondroitinase ABC, has been found to reduce the firing threshold of these cells *in vitro* (Dityatev et al., 2007), which would promote rather than deter GABAergic inhibition.

Other possible mechanisms may be related to alterations of the perisynaptic ECM by Hyase. An elegant study by Frischknecht et al. (2009) used Hyase or chondroitinase ABC to remove PNNs from cultured rat hippocampal neurons and revealed that a perisynaptic net-like structure in the ECM influences the mobility of AMPA receptors, creating a barrier for their movement into and out of excitatory synapses. ECM removal impairs paired-pulse depression of excitatory postsynaptic currents (EPSCs) in cultures and thus might promote hyperexcitation in cultured pyramidal cells (Frischknecht et al., 2009).

Our MEA recordings revealed that the spontaneous electrical activity of mature dissociated hippocampal neurons grown on MEAs occurs in the form of synchronized burst discharges, but the pattern of these burst discharges is different from the long-lasting “superburst” discharges induced by Hyase treatment.****These****“superbursts” became most prominent 9 days after the Hyase treatment. Our Ca^2+^ imaging data demonstrates that the Hyase treatment also transformed the normal neuronal Ca^2+^ oscillations into long-lasting “superoscillations” with mean durations of up to 100 s already on the 2nd day after the Hyase treatment. Thus, dramatic changes in Ca^2+^ oscillations precede changes in spiking activity, suggesting that superoscillations may represent a key event leading to changes in the expression of genes that determine neuronal excitability. In addition, changes in Ca^2+^ signaling in astrocytes were detected after the Hyase treatment, although at a smaller scale than in neurons.

Elevations in burst activity and neuronal Ca^2+^ transients in the Hyase-treated neurons and astrocytes was suppressed by an application of the L-VDCC blocker diltiazem, although not by a NMDA receptor antagonist, whereas the superbursts of spiking activity were additionally suppressed by CNQX, an antagonist of AMPA receptors. The latter is not surprising because CNQX mostly blocks excitatory transmission. The elimination of superbursts and superoscillations by diltiazem is at first glance surprising because HA was reported to support L-VDCC activity and the Hyase treatment leads to an acute deficit in neuronal L-VDCC-mediated currents (Kochlamazashvili et al., 2010). It is plausible to assume that this acute deficit in L-VDCC activity might be later overcompensated by overexpression of L-VDCCs after the Hyase treatment, which might lead to the generation of Ca^2+^ superoscillations and downstream effects on neuronal excitability/excitatory transmission. This hypothesis remains to be verified in follow-up studies. The presented experimental data support the view that the remodeling of ECM may lead to generation of epileptiform activity *in vitro* in the form of superbursts. Such epileptogenic degradation of HA and ECM remodeling may be due to genetic factors or triggered by insults such as stroke or a brain injury. Thus the described experimental model could be potentially valuable for development of new class of broadly applicable anti-epileptogenic drugs, which would target the HA-based neural ECM and/or ECM-mediated signaling.

## Conflict of Interest Statement

The authors declare that the research was conducted in the absence of any commercial or financial relationships that could be construed as a potential conflict of interest.
